# Supplementary material for: Evidence for preferred propagating terrestrial heatwave pathways due to Rossby wave activity
Source: Nat Commun. 2025 May 22;16:4742. doi: 10.1038/s41467-025-60104-w (PMC12095606; doi:10.1038/s41467-025-60104-w)
Supplement: Supplementary file 1 — Supplementary Information [file 41467_2025_60104_MOESM1_ESM.pdf]

Supplementary Information for

Evidence for preferred propagating terrestrial heatwave pathways due to Rossby wave activity

Mingzhao Wang[1,2+], Yu Huang[2,3+*], Christian L. E. Franzke[4,5], Naiming Yuan[6,7,8*], Zuntao Fu[1*], Niklas Boers[2,3]

[1]Laboratory for Climate and Ocean-Atmosphere Studies, Department of Atmospheric and Oceanic Sciences, School of Physics, Peking University, Beijing, China.
[2]Earth System Modelling, School of Engineering and Design, Technical University of Munich, Munich, Germany.
[3]Complexity Science, Potsdam Institute for Climate Impact Research, Potsdam, Germany.
[4]Center for Climate Physics, Institute for Basic Science, Busan, Republic of Korea.
[5]Department of Integrated Climate System Science, Pusan National University, Busan, Republic of Korea.
[6]School of Atmospheric Sciences, Sun Yat-sen University, Zhuhai, China.
[7]Key Laboratory of Tropical Atmosphere-Ocean System, Ministry of Education, Zhuhai, China.
[8]Southern Marine Science and Engineering Guangdong Laboratory, Zhuhai, China.
[+]M.W. and Y.H. contributed equally to this work.

*Corresponding author(s). E-mail(s): y.huang@tum.de; yuannm@mail.sysu.edu.cn; fuzt@pku.edu.cn;

Contents

Supplementary Figures 3

Supplementary Notes 35

Supplementary Figures

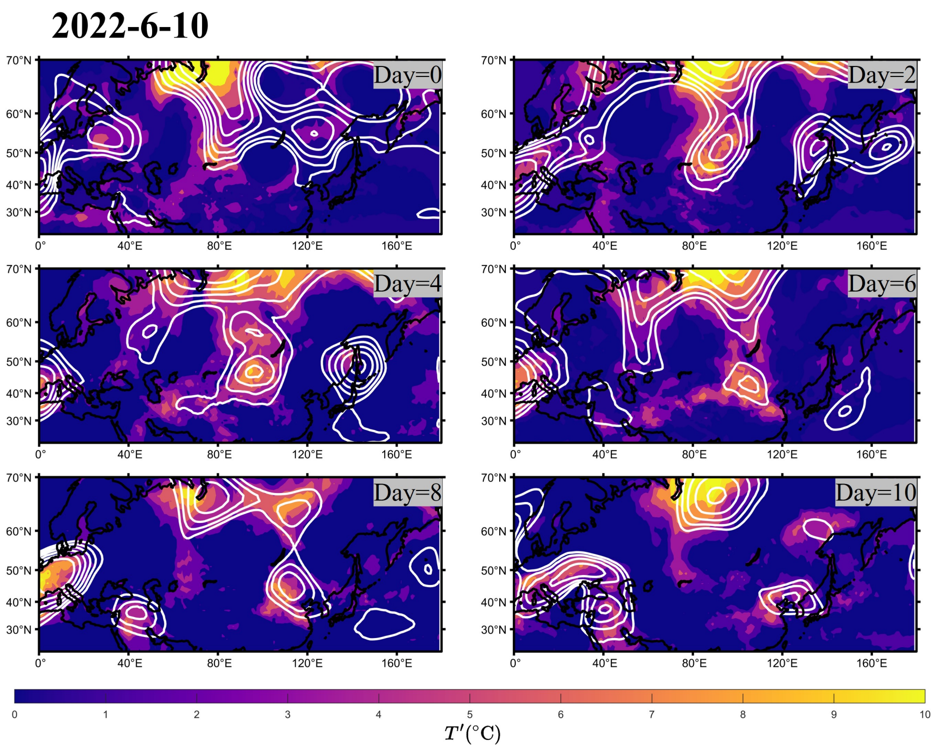

Fig. S1 On June 10, 2022, the distribution of surface temperature and 500 hPa geopotential height anomalies (contours) across Asia. Days 0 to 10 represent the evolution of surface temperature anomalies from the onset of the heatwave to 10 days thereafter, illustrating a complete process of heatwave propagation from the Ural to the south and middle of East Asia.

1995-5-2

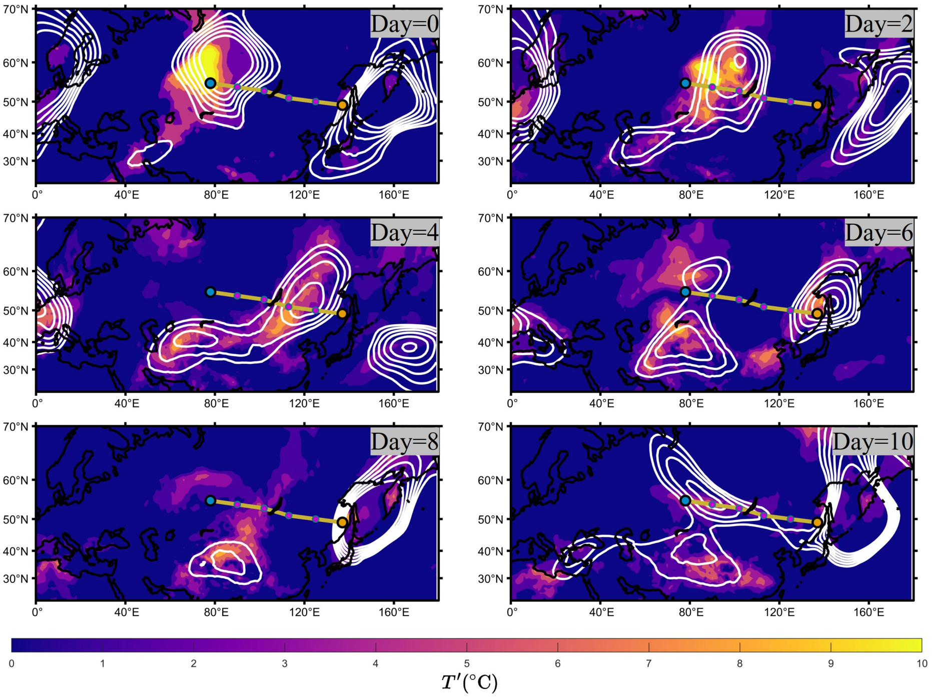

Fig. S2 On May 2, 1995, the distribution of surface temperature and 500 hPa geopotential height anomalies (contours) across Asia. Days 0 to 10 represent the evolution of surface temperature anomalies from the onset of the heatwave to 10 days thereafter, illustrating a complete process of heatwave propagation from the Urals to East Asia. This aligns with the propagation pathways discovered through local searching algorithm (indicated in the figure).

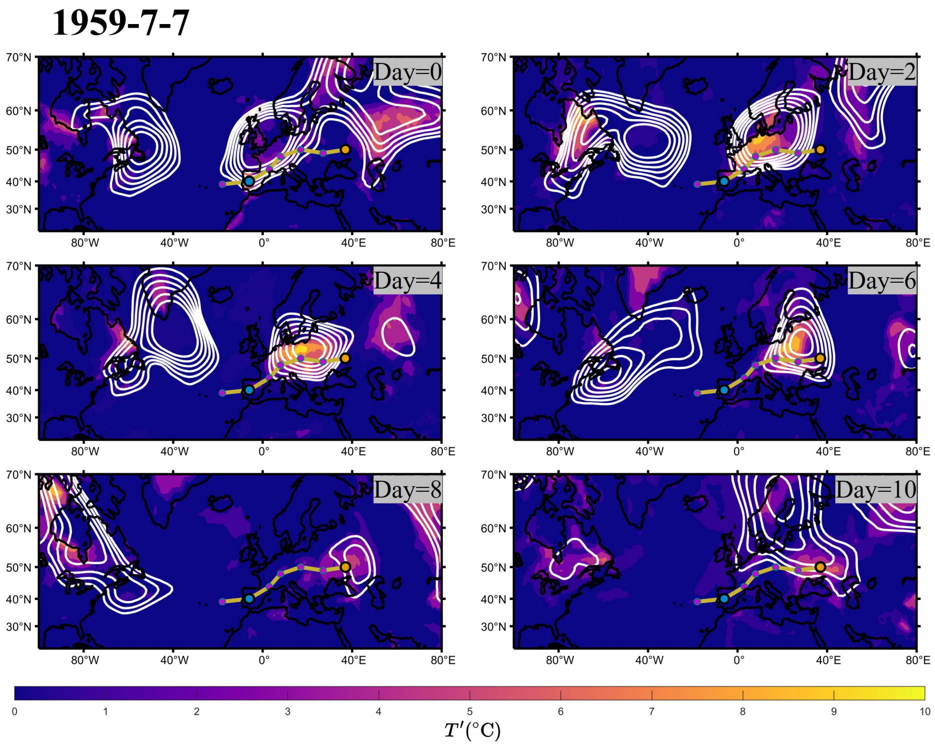

Fig. S3 Similar to Fig.S2, but shows the distribution of surface temperature anomalies across West Europe on July 7, 1959, corresponding to the propagation pathway of Western Europe.

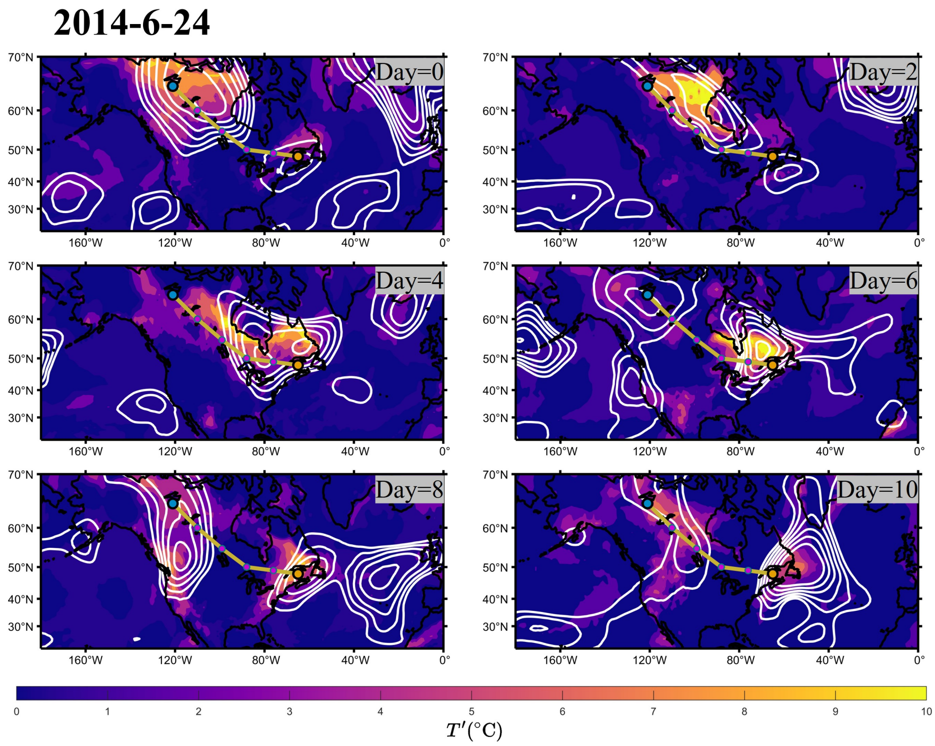

Fig. S4 Similar to Fig.S2, but shows the distribution of surface temperature anomalies across North America on June 24, 2014, corresponding to the propagation pathway of North America 1.

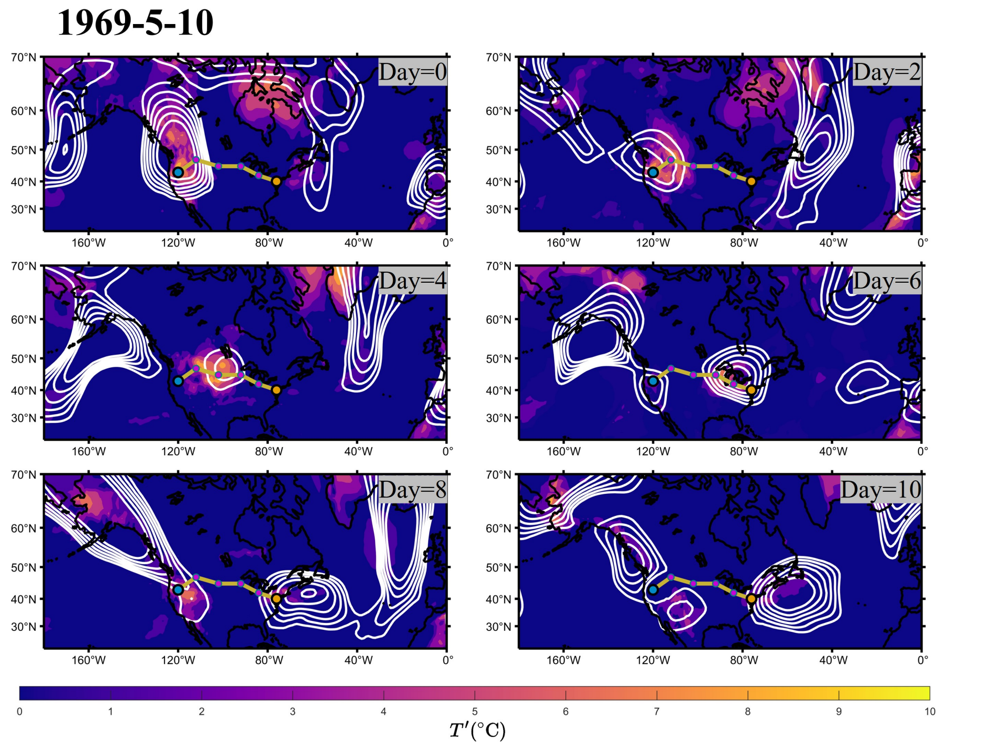

Fig. S5 Similar to Fig.S2, but shows the distribution of surface temperature anomalies across North America on May 10, 1969, corresponding to the propagation pathway of North America 2.

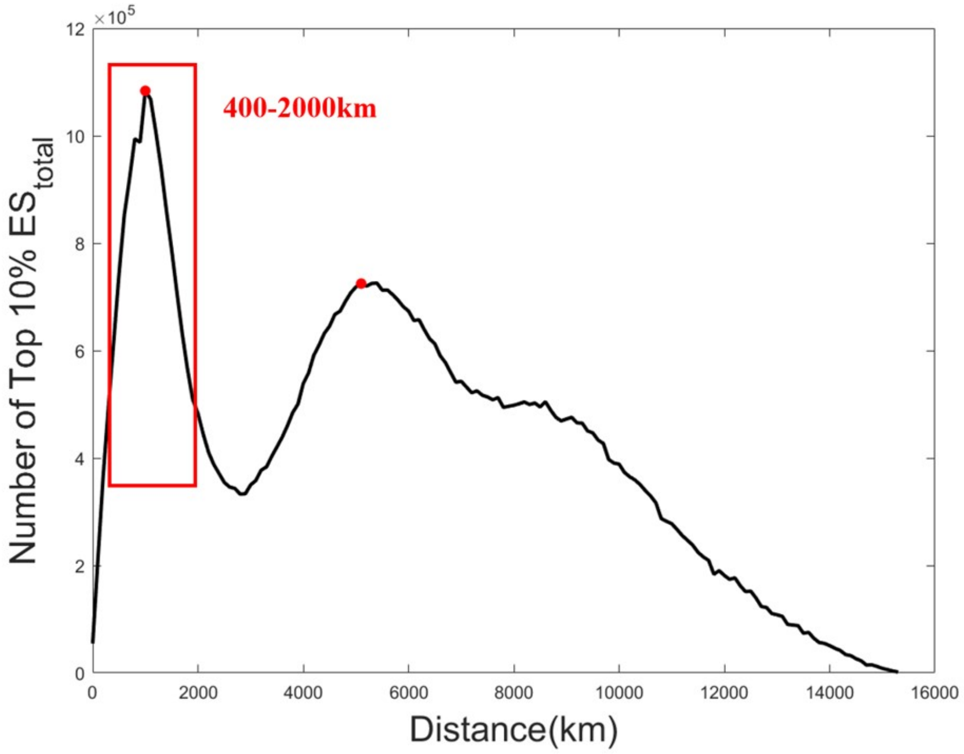

Fig. S6 Distribution of the top 10% $\mathrm{ES}_{\mathrm{total}}$ counts with respect to the geographical distance of edges, where $\mathrm{ES}_{\mathrm{total}}$ includes three parts between grid cells i and j: i occurring before j, j occurring before i, and simultaneous occurrences between them. The distribution shows two distinct peak positions at distances s_1 = 1000km and s_2 = 5100km. These peaks represent the regional propagation scale and the teleconnected scale of heatwaves, respectively. A distance range of 400-2000km has been selected for further research on the propagation of heatwaves, which encompasses more than 95% of the propagation events.

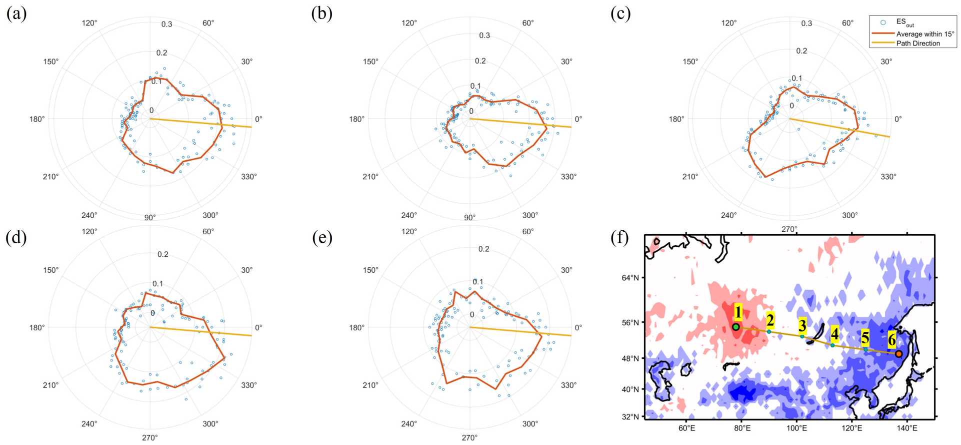

Fig. S7 Rose diagrams for directional distribution of ES strengths [1, 2] (Asia pathway). (a) shows the distribution of ES_{out} values within a radius of 800-1200km centered at the starting node (pathway node 1) according to direction (blue dots), with the red line representing the average value within every 15° azimuth, and the yellow line indicating the direction from the starting node to pathway node 1. (b)-(e) are similar to (a) but are centered at pathway nodes 2, 3, 4, and 5 respectively, with the yellow line indicating the direction to the next pathway node from the center. (f) depicts the positions of the corresponding pathways and the network divergence of the respective regions.

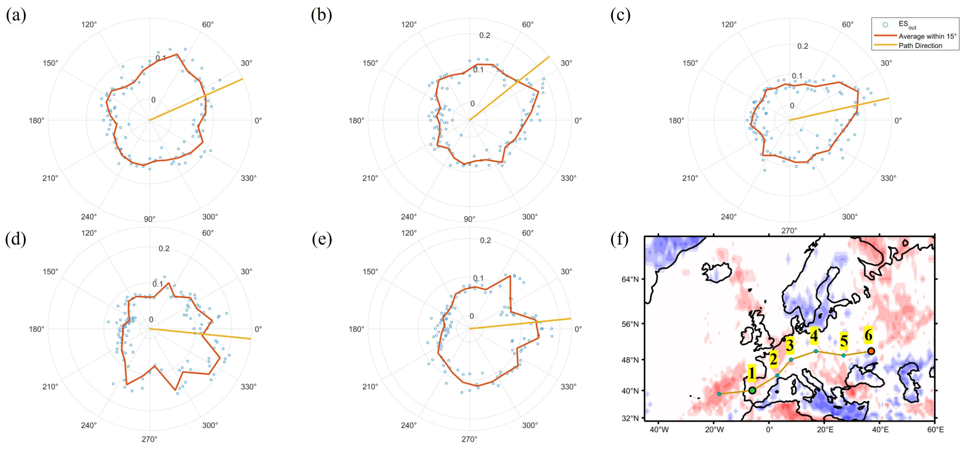

Fig. S8 Similar to Fig.S7, but shows ES rose diagrams for the pathway of West Europe.

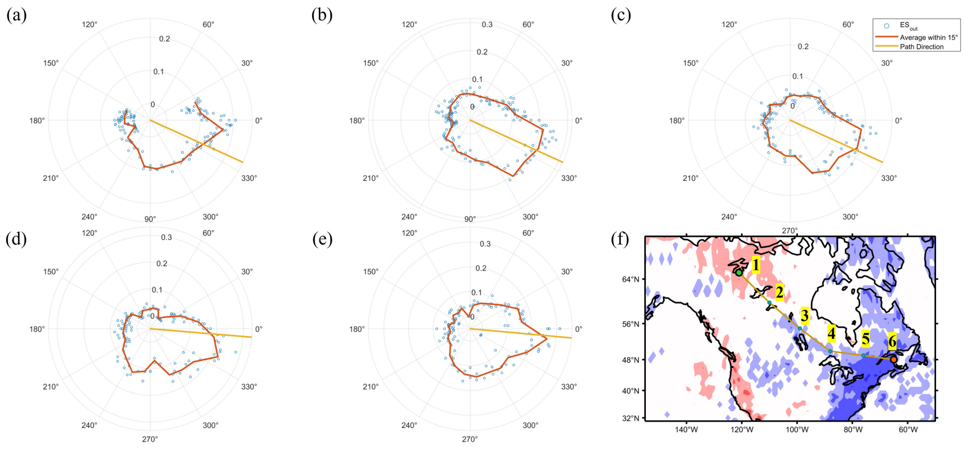

Fig. S9 Similar to Fig.S7, but shows ES rose diagrams for the pathway of North America 1.

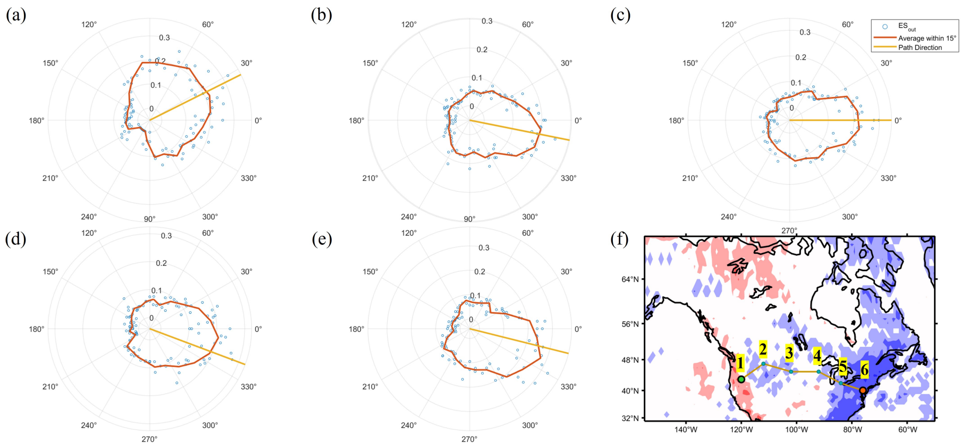

Fig. S10 Similar to Fig.S7, but shows ES rose diagrams for the pathway of North America 2.

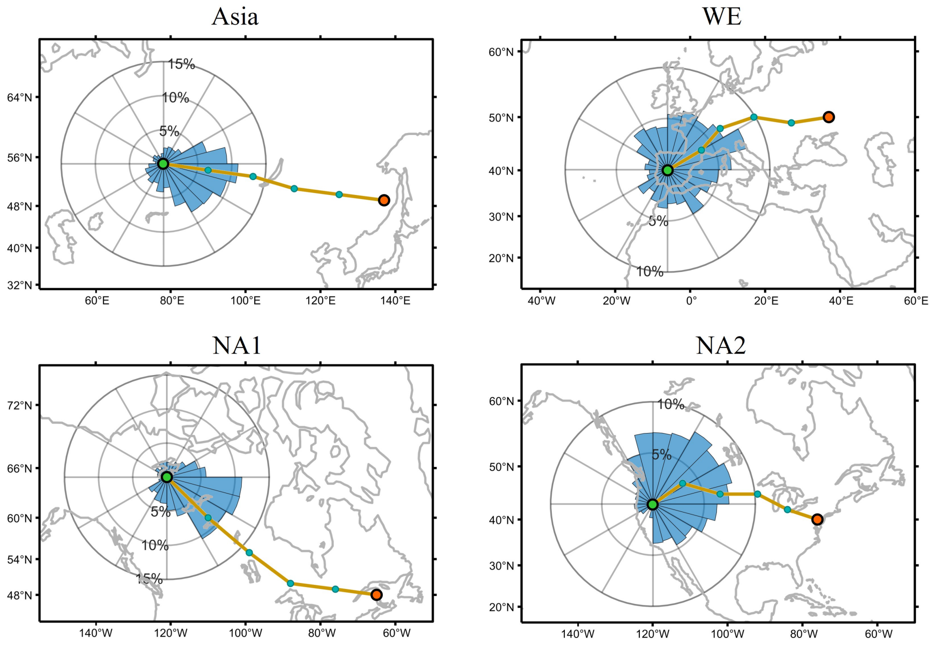

Fig. S11 Rose diagrams of movement directions for the heatwaves at starting nodes of the four preferred pathways (Asia, WE, NA1 and NA2 pathways), respectively. The dots connected with yellow lines denote the spatial routes of the four preferred pathways.

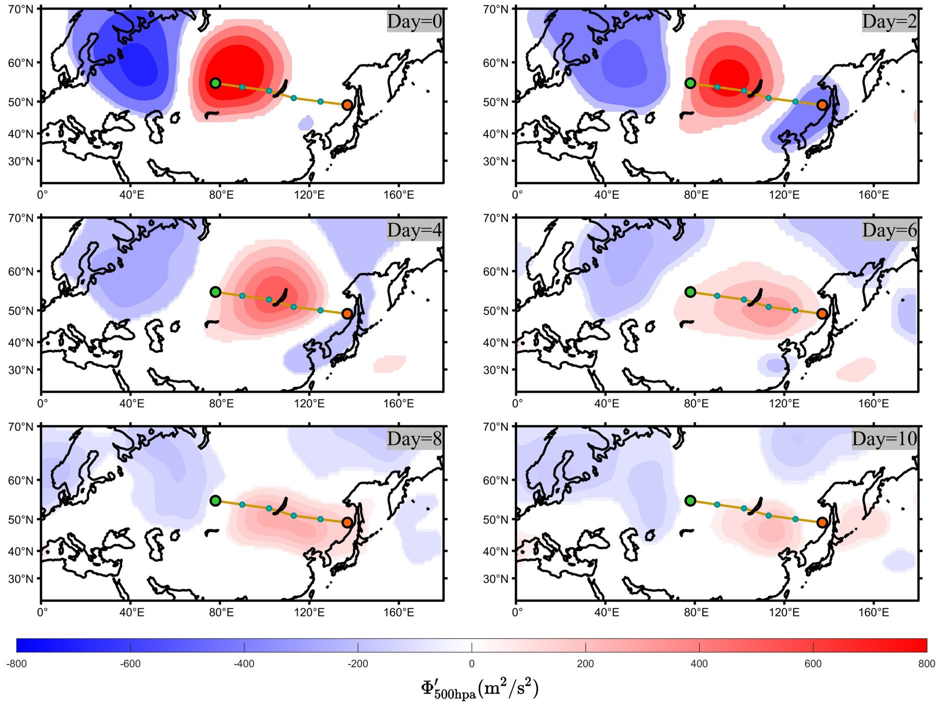

Fig. S12 The composite charts of gravitational potential anomalies at 500 hPa for Asia after a heatwave occurs at the starting node. Day = 0 to 10 represent the changes in gravitational potential from the start of the heatwave to the 10th day after its occurrence. Only values significant at the 5% significance level are displayed.

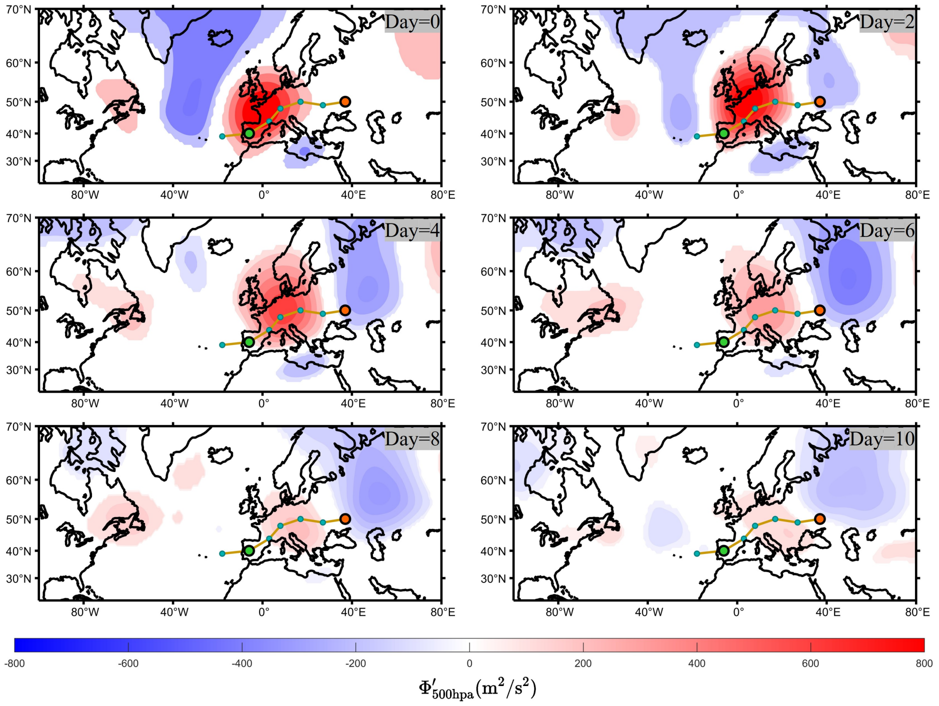

Fig. S13 Similar to Fig.S12, but a composite for the pathway of West Europe.

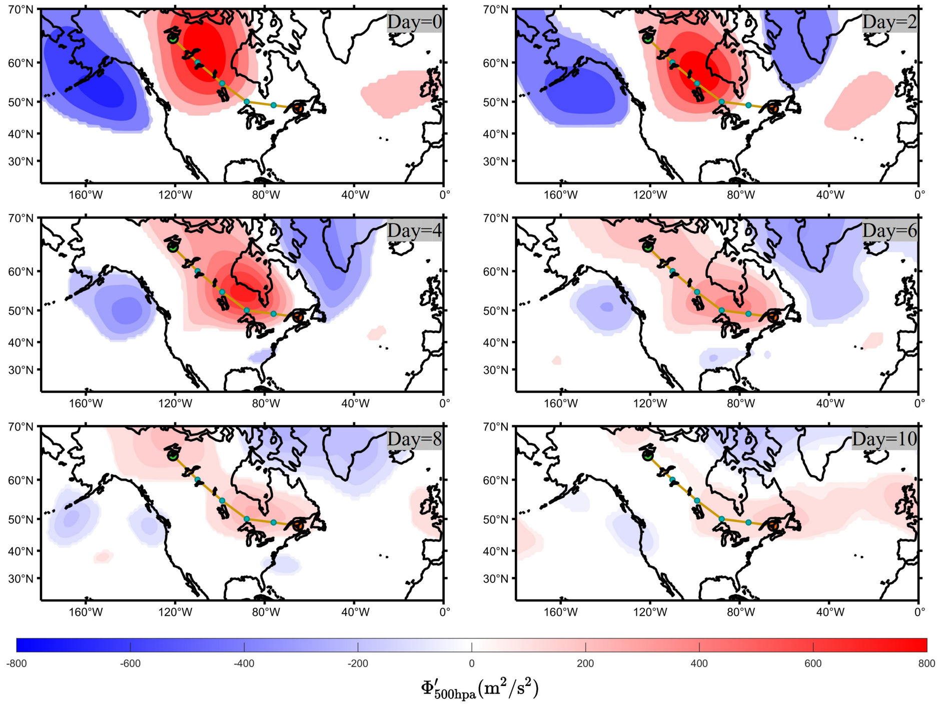

Fig. S14 Similar to Fig.S12, but a composite for the pathway of North America 1.

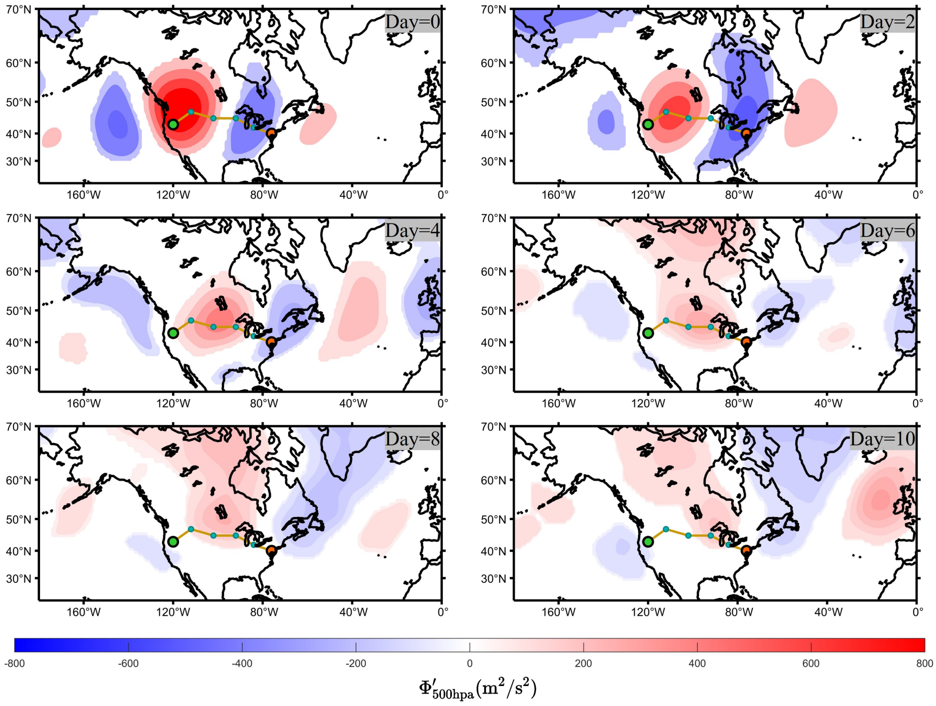

Fig. S15 Similar to Fig.S12, but a composite for the pathway of North America 2.

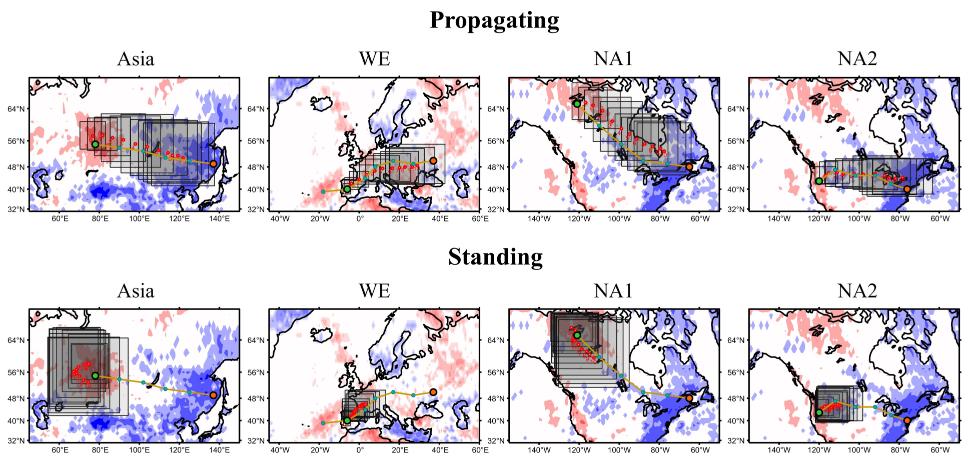

Fig. S16 Similar to Fig.2 in the main text, but clustering is based on high-value centers of surface temperature anomalies, which can also be categorized into propagating/standing types, closely matching the results of Z500 anomalies.

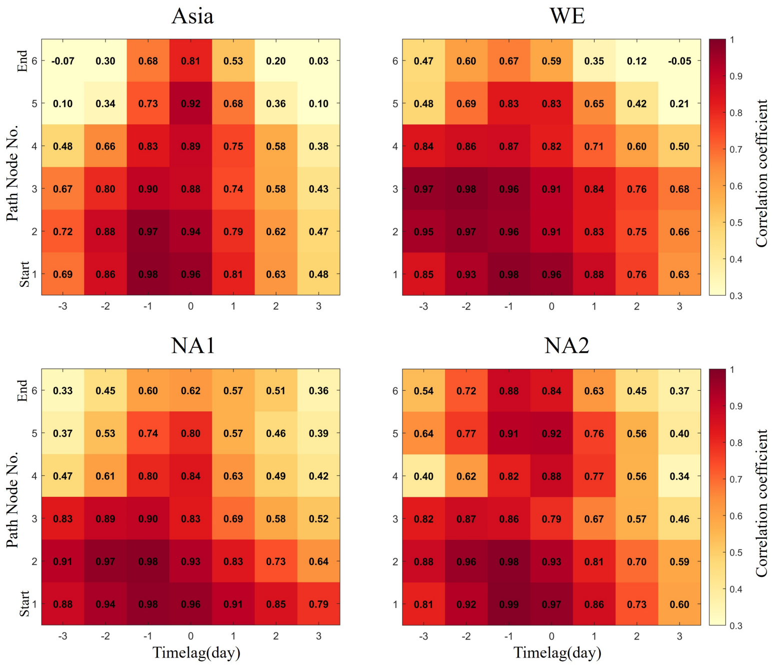

Fig. S17 Time-lagged Pearson correlation between temperature and Z500 anomalies at individual path nodes during periods of propagating heatwaves, with results presented for the Asia, WE, NA1, and NA2 heatwave pathways, respectively. Positive time lag indicates that the Z500 anomaly is lagged relative to the temperature anomaly. Only cases of propagating heatwaves are included in this analysis. The Z500 anomaly is highly correlated (with Pearson correlation higher than 0.8) with the temperature anomaly at time lag = 0, and this holds for all path nodes. Specifically, at time lag = -1, where the Z500 anomaly precedes the temperature anomaly by 1 day, the first three path nodes exhibit a maximum correlation (higher than 0.94) between the temperature and Z500 anomalies, indicating that the Z500 anomaly leads the temperature anomaly.

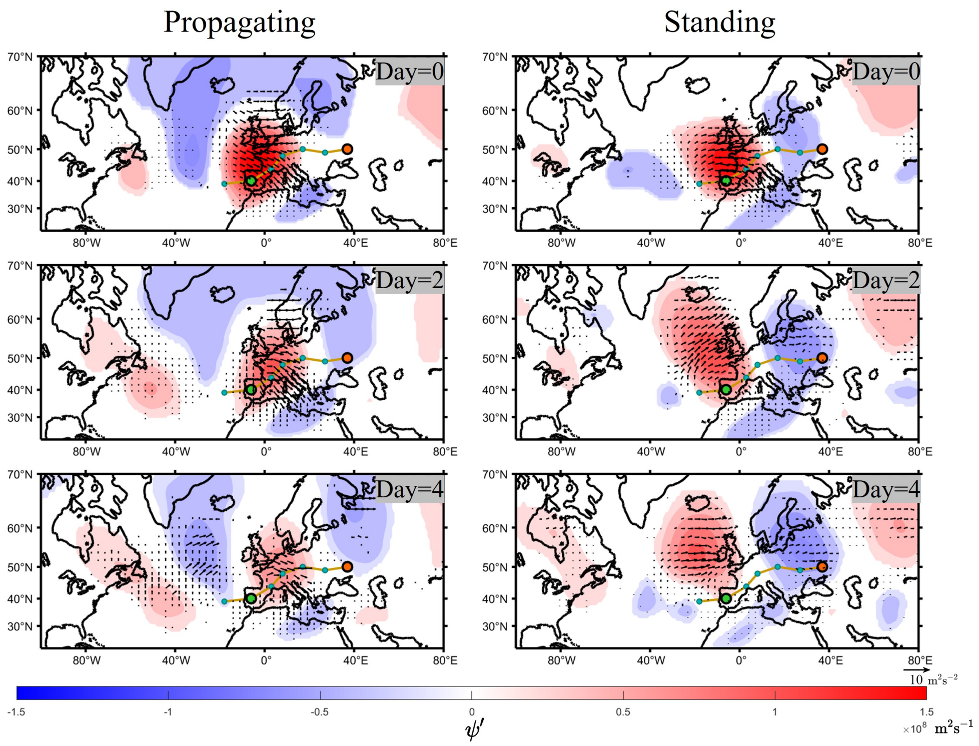

Fig. S18 Similar to Fig.3 in the main text, the composite charts of the perturbed stream function field (color shading) and horizontal TN flux (arrows) at 300 hPa for West Europe after a heatwave occurs at the starting node. Day = 0, 2, and 4 represent the central time of the heatwave occurrence at the starting node and the subsequent 2 days and 4 days, respectively. Only values significant at the 5% significance level are displayed.

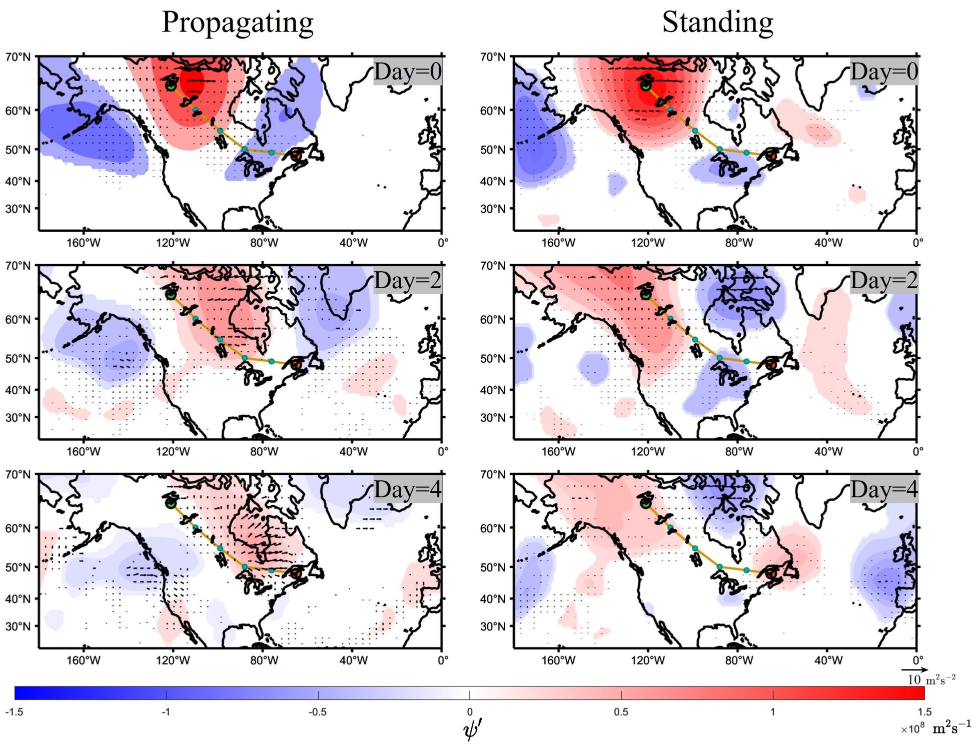

Fig. S19 Similar to Fig.S18, but for the region of North America 1.

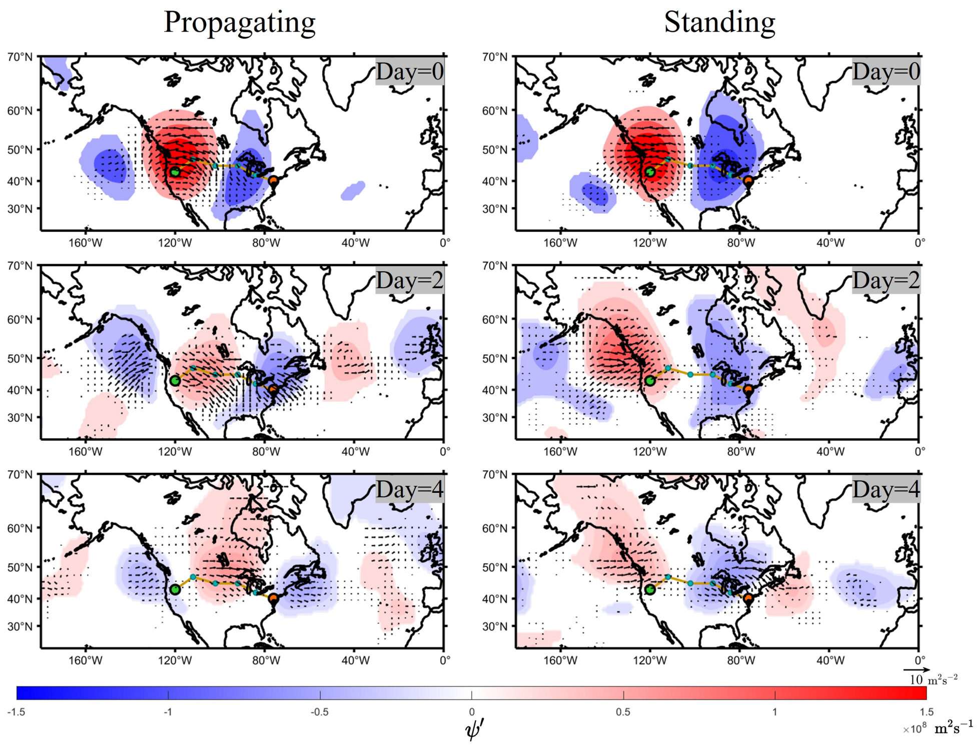

Fig. S20 Similar to Fig.S18, but for the region of North America 2.

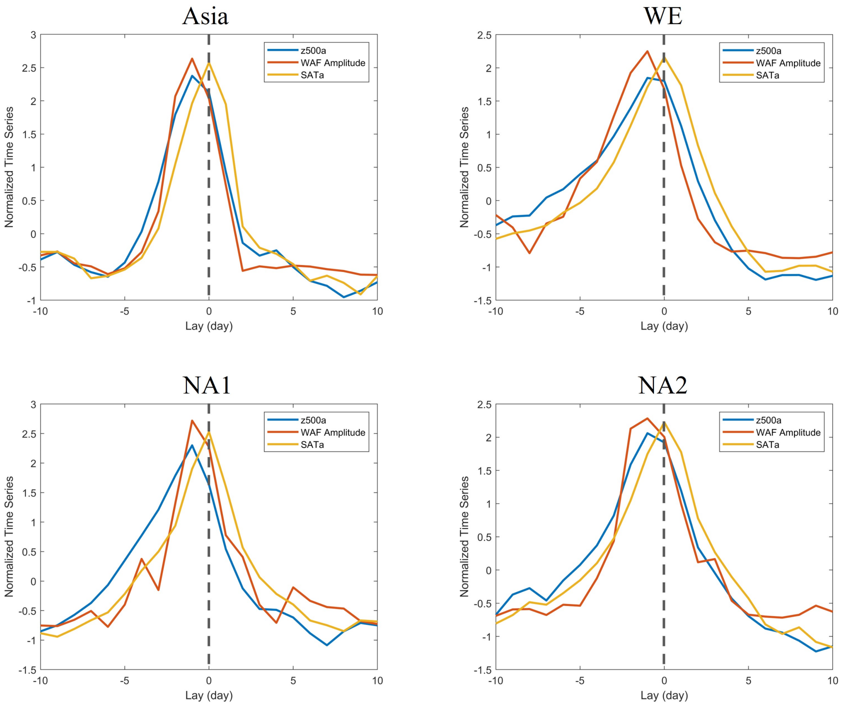

Fig. S21 Comparison of composite time series during propagating heatwaves for the geopotential height anomaly at 500 hPa (z500), the absolute amplitude of TN flux for Rossby wave activity (WAF amplitude), and surface air temperature anomaly (SATa) at the starting nodes of the Asia, WE, NA1, and NA2 pathways, respectively. Time = 0 corresponds to the timing of the local temperature maximum during the heatwave at the starting node. To facilitate comparison, the time series of each variable were normalized to have a mean of 0 and a standard deviation of 1.

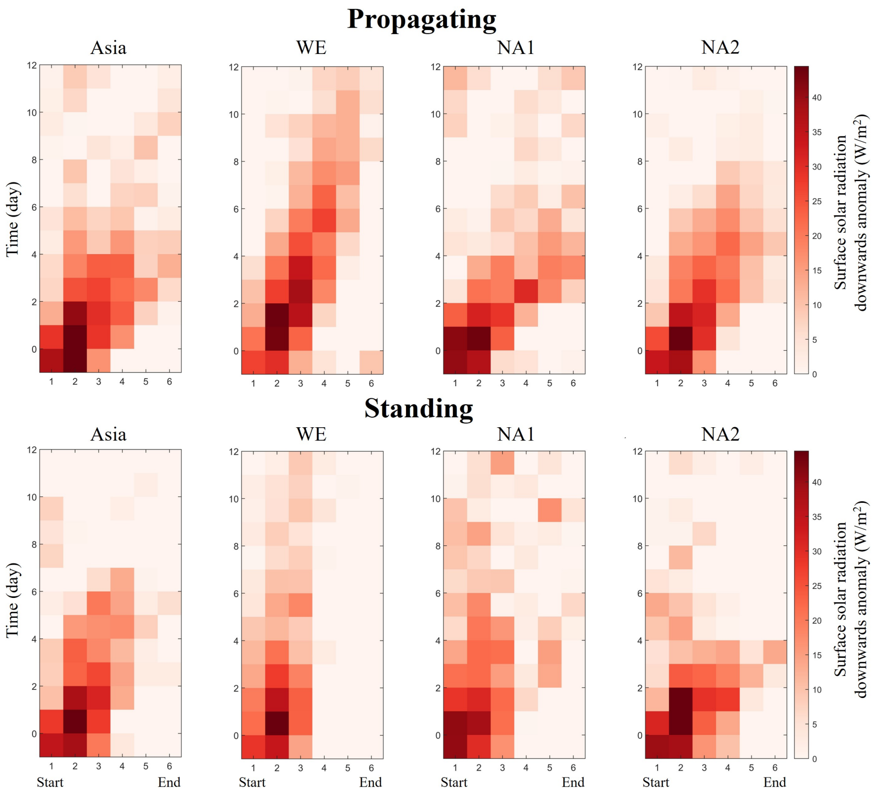

Fig. S22 Spatiotemporal evolutions of surface solar radiation anomaly during propagating and standing heatwaves. Composite surface solar radiation anomaly as a function of time and the spatial nodes on the detected propagation pathways. Horizontal axis denotes the six representative nodes on the four detected propagation pathways (see Fig. 1). Time = 0 corresponds to the timing of the local temperature maximum during the heatwave at the starting node.

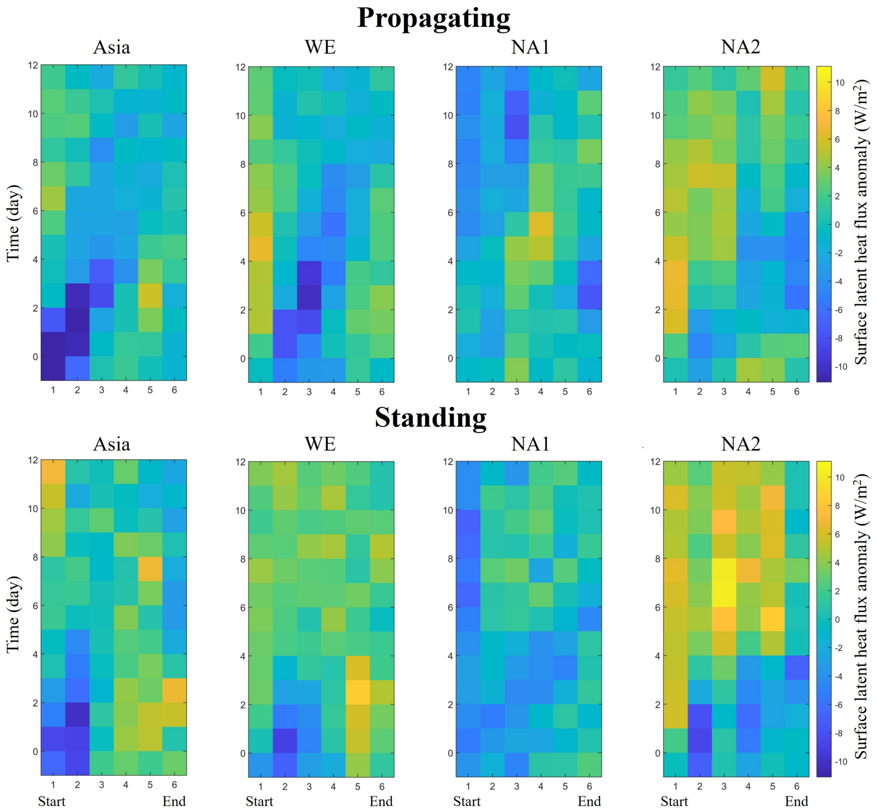

Fig. S23 Spatiotemporal evolutions of surface latent heat flux anomalies during propagating and standing heatwaves. Composite surface latent heat flux anomaly as a function of time and the spatial nodes on the four detected propagation pathways. The horizontal axis denotes the six representative nodes on the detected propagation pathways (see Fig. 1). Time = 0 corresponds to the timing of the temperature's local maximum during the heatwave at the starting node.

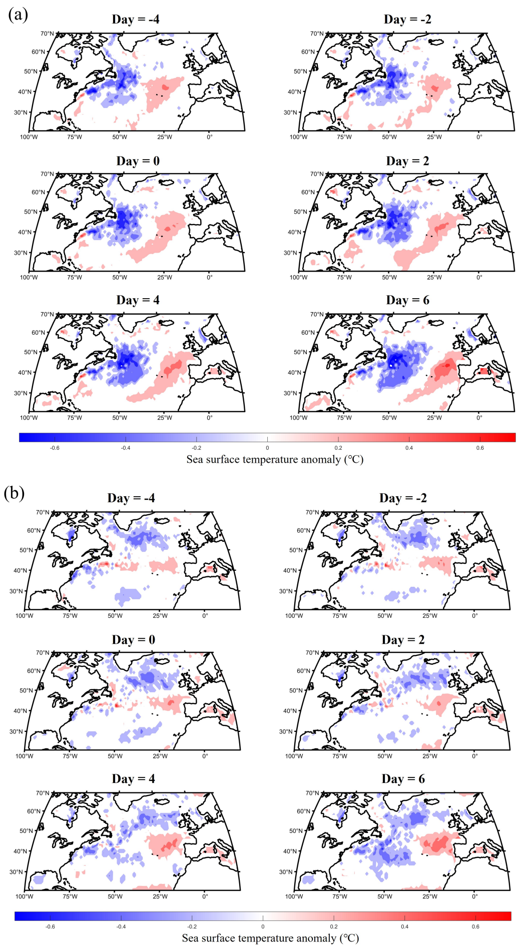

Fig. S24 Composite sea surface temperature anomaly during (a) the propagating heatwaves and (b) the standing heatwaves on the Western Europe pathway. Only values significant at the 5% significance level are displayed.

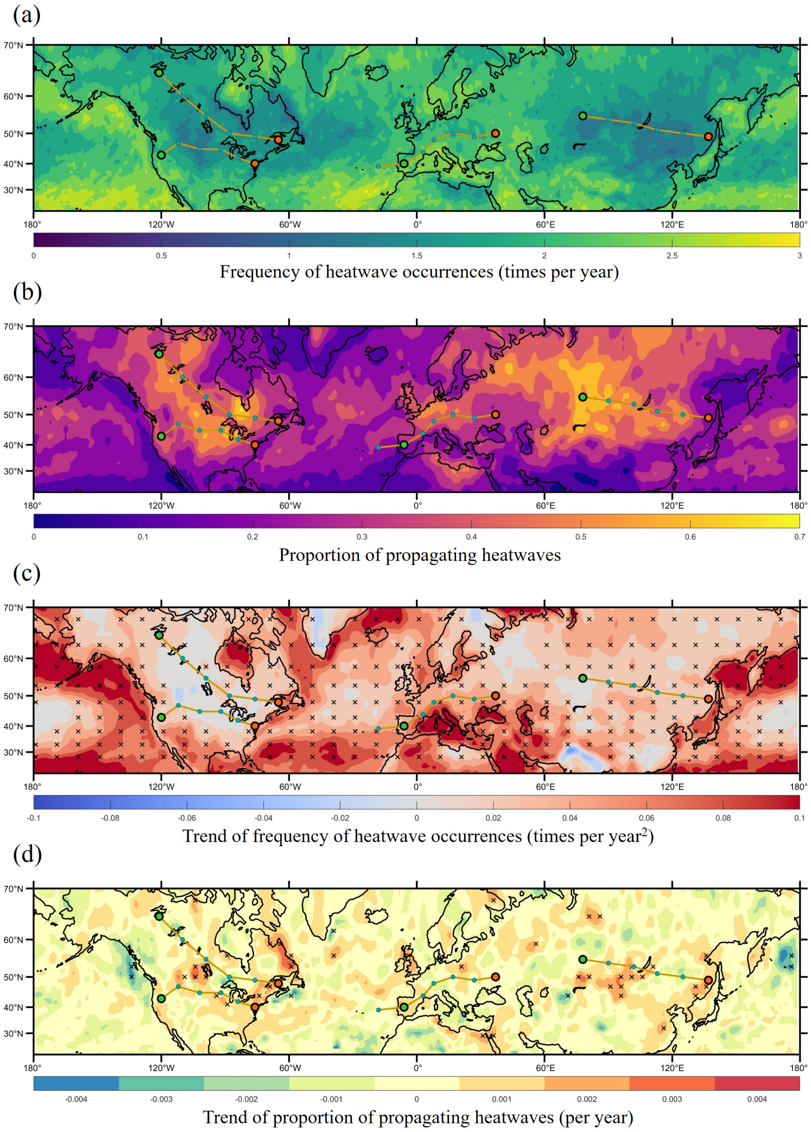

Fig. S25 Statistics of heatwave occurrences (1959-2023) in the Northern Hemisphere from 20°N to 70°N, and comparison with the preferred propagation pathways of heatwaves revealed in our study (yellow lines). (a) Frequency of heatwave occurrences; (b) proportion of the cases of propagating heatwaves (Supplementary Note 1); (c) trend of the frequency of heatwave occurrences; and (d) trend of the proportion of the propagating heatwaves. Cross marks indicate trend values that are significant at the 5% significance level.

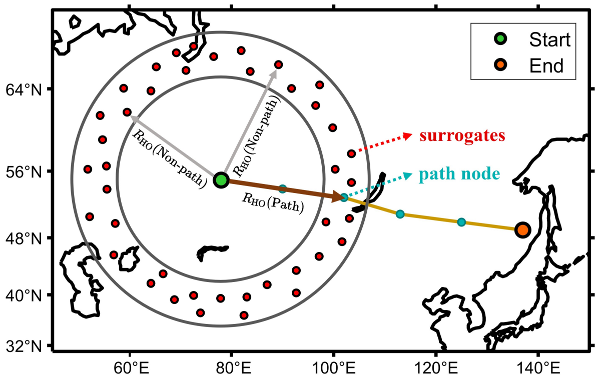

Fig. S26 Illustration of surrogate generation for the R_{HO} significance test. Taking the 3rd path node of the Asia pathway as an example, we consider all grid cells at a similar distance from the starting node, comparable to the distance between the starting node and the 3rd path node (around 1600 km). These grid cells, represented by the red dots within black rings with radii of 1400 km and 1800 km, are not part of the Asia pathway but serve as surrogates for the 3rd path node in subsequent significance tests. The brown arrow indicates that the R_{HO} calculation is performed on the 3rd path node of the Asia pathway, and the result is denoted as R_{HO}(Path). The gray arrow, instead, indicates that the R_{HO} calculation is performed on the surrogates, denoted as R_{HO}(Non-path). See Supplementary Note 4 for the details of calculating R_{HO}.

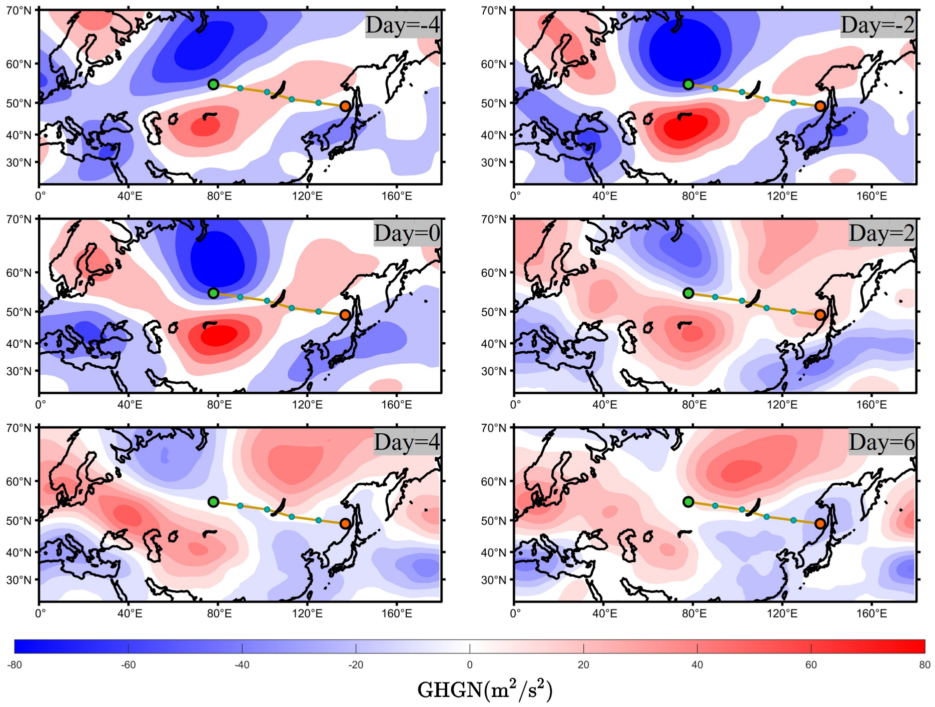

Fig. S27 Spatiotemporal distribution of GHGN blocking index (Supplementary Note 5) for the standing heatwave patterns in the Asia pathway. Day = 0, 2, and 4 represent the the timing of the local temperature maximum during the heatwave at the starting node, and the subsequent 2 and 4 days, respectively. Day= -4 and -2 denote the 2 and 4 days before the timing of the local temperature maximum during the heatwave. A more negative GHGN value indicates a stronger blocking.

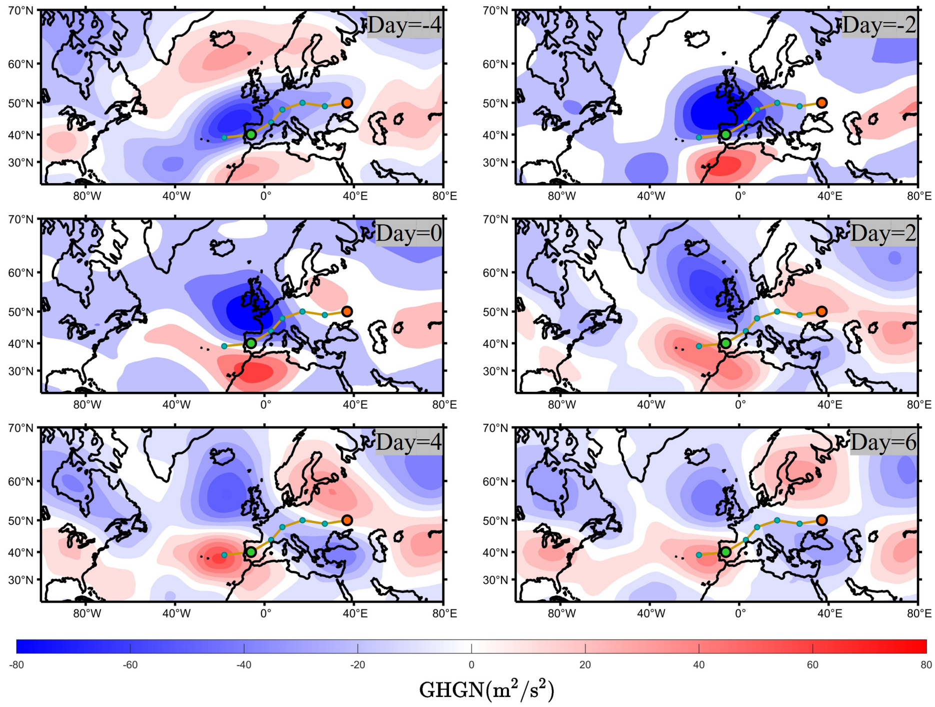

Fig. S28 Similar to Fig.S27, but for the standing heatwave patterns in the WE pathway.

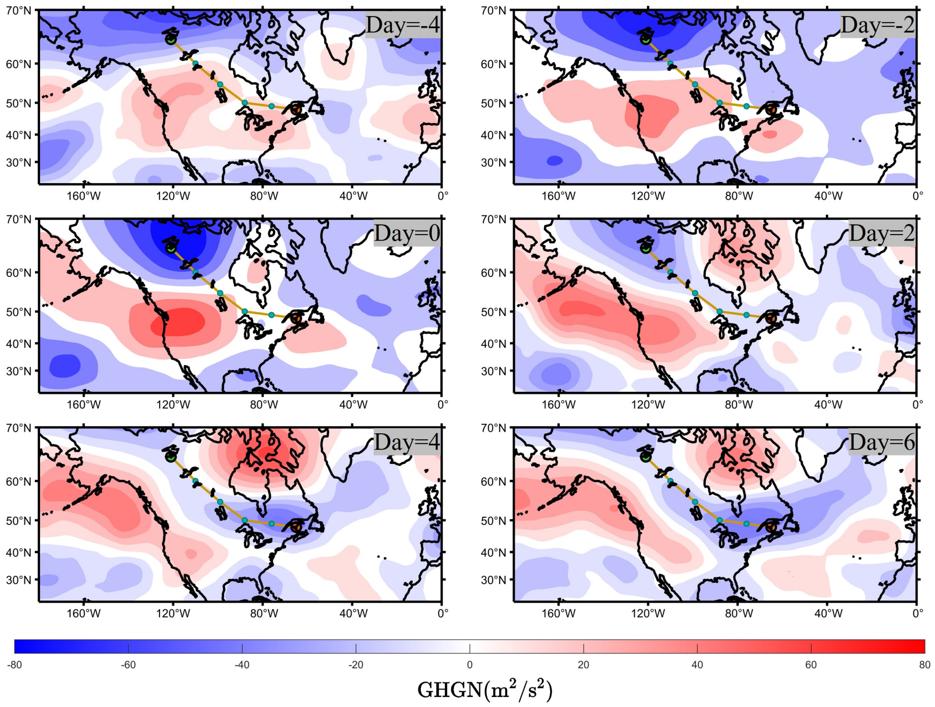

Fig. S29 Similar to Fig.S27, but for the standing heatwave patterns in the NA1 pathway.

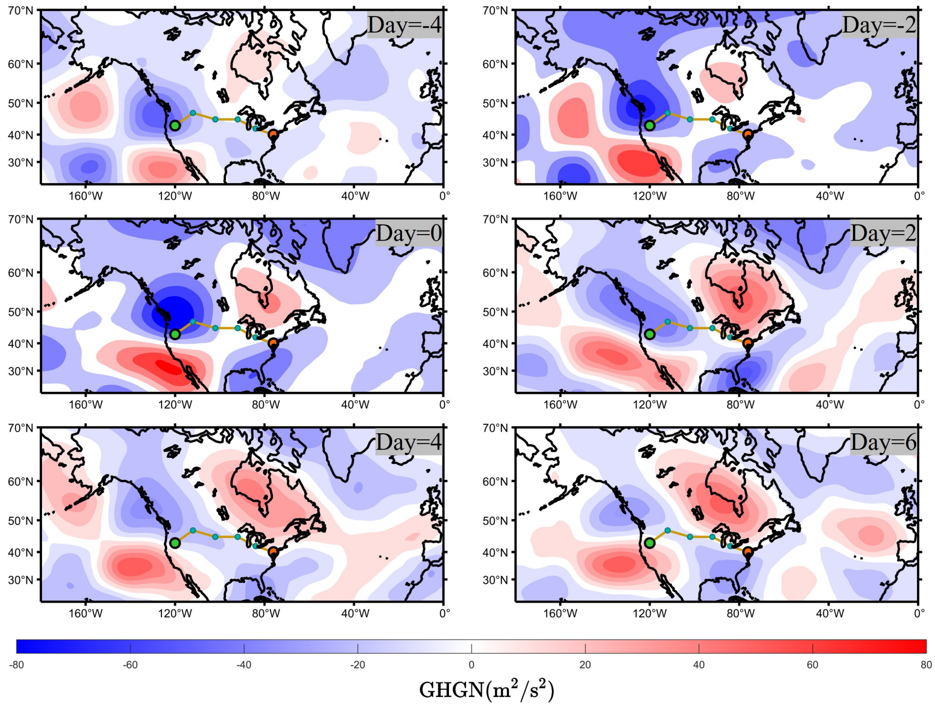

Fig. S30 Similar to Fig.S27, but for the standing heatwave patterns in the NA2 pathway.

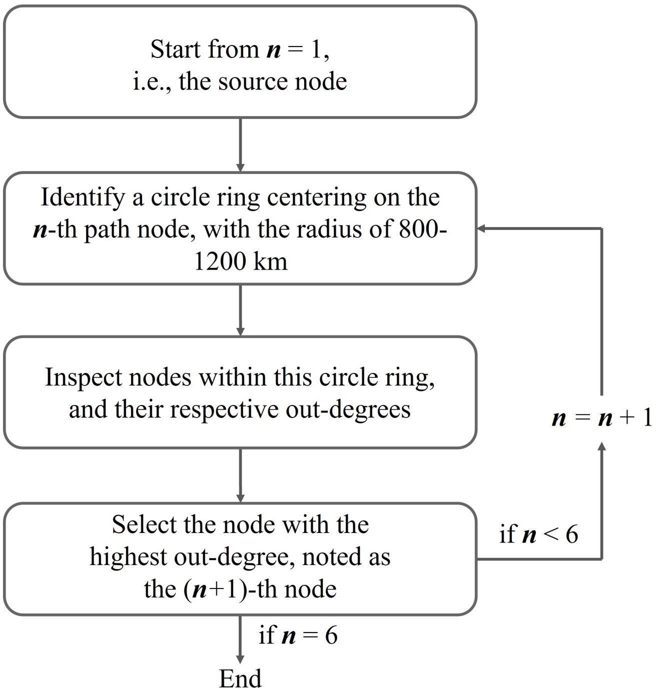

Fig. S31 Flowchart diagram illustrating the local searching algorithm for identifying preferred propagation pathway of heatwaves in the complex network.

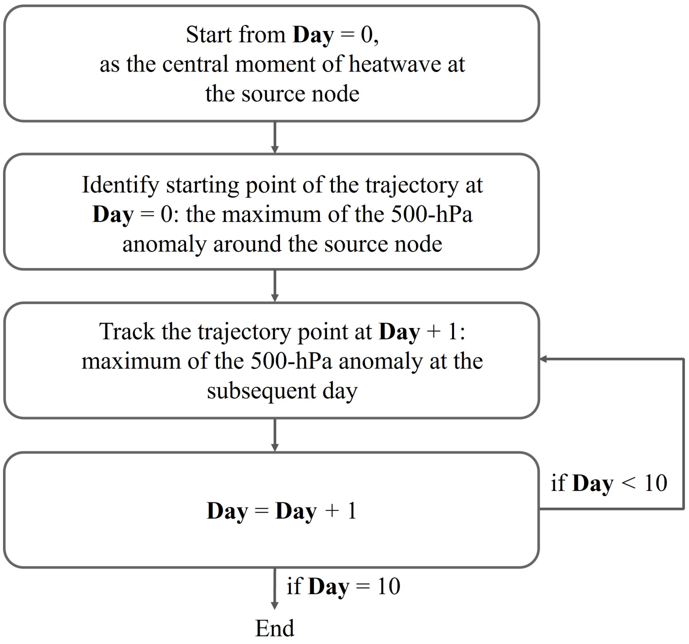

Fig. S32 Flowchart diagram illustrating the trajectory tracking algorithm for determining the movement trajectory of a high-pressure system over a certain region following the occurrence of a heatwave. Central moment of the heatwave denote the timing of the local temperature maximum during the heatwave at the source node.

Supplementary Notes

Supplementary Note 1: Estimating the proportion of propagating heatwaves using complex network

As outlined in the Methods section, the complex network for heatwaves enables counting the number of heatwave events propagating from an interested spatial grid cell to another grid cell. Thus, for heatwaves in a given spatial grid cell i, we can estimate the proportion of the cases of propagating heatwaves as follows.

Considering another spatial grid cell j whose distance from i is between 400 and 2000 km (Fig.S6), we take e_i^μ as the timing of the local temperature maximum during the μ-th heatwave event at grid cell i, and e_j^v is for grid cell j, where $\mu, v \in [1, l]$, l denotes the total number of events at each grid cell. Then we calculate the dynamical delay $d_{ji}^{v,\mu} := e_j^v - e_i^\mu$, and the threshold τ:

$$\tau = \min \left(\frac{\left\{ d_{jj}^{v,v-1}, d_{jj}^{v,v+1}, d_{ii}^{\mu,\mu-1}, d_{ii}^{\mu,\mu+1} \right\}}{2} \right) \quad (1)$$

Specifically for the μ-th heatwave event at the interested grid cell i, this event is counted as a propagating case if there exists at least one grid cell j such that $0 < d_{ji}^{v,\mu} \leq \tau$. This examination is performed for all heatwave events at the interested grid cell i, and the proportion of propagating cases is then calculated. Fig.S25b shows the proportion of propagating heatwave cases at various spatial grid cells in the Northern Hemisphere from 20°N to 70°N.

Supplementary Note 2: Identifying trajectory and uncertainty of the high-pressure system movement

Figure 2 presents the trajectory and uncertainty of the high-pressure system. Taking the Asia pathway as an example, our procedure is as following steps:

(1) For each heatwave event, we first identify the geographic location with the maximum of the 500-hPa anomaly around the starting node (on Day = 0, i.e., the timing of the local temperature maximum during the heatwave at the starting node), designating this as the center of the high-pressure system on Day = 0.

(2) For the subsequent days, we identify the centers of the high-pressure system from Day 1 to Day= 10, allowing us to track the movement of the high-pressure system throughout the heatwave event.

(3) This process yields the ensemble movement trajectories for all heatwave events, and we apply k-means clustering to categorize them into propagating and standing patterns.

(4) For each day's set of trajectory points, we calculate the average geographic location as the center. The uncertainty for each point is determined as twice the standard deviation of its latitude and longitude, as shown in Fig. 2.

Supplementary Note 3: A causality analysis on the relationship between the heatwave and the RWP in the propagation pathway

Granger causality is a statistical hypothesis test used to determine whether one time series can predict another [3, 4]. It is based on the premise that if a variable X "Granger-causes" another variable Y, then past values of X contain information that helps predict future values of Y, beyond what is already contained in past values of Y alone. The Granger causality test is based on vector autoregressive models. Consider two time series X_t and Y_t. The standard autoregressive representation of Y_t is:

$$Y_t = \sum_{i=1}^{p} \alpha_i Y_{t-i} + \varepsilon_t, \tag{2}$$

where ε_t is a white noise error term and p is the number of lags. To test whether X_t Granger-causes Y_t, we extend the model to include past values of X_t:

$$Y_t = \sum_{i=1}^{p} \alpha_i Y_{t-i} + \sum_{j=1}^{q} \beta_j X_{t-j} + \varepsilon_t. \tag{3}$$

If the coefficients β_j are jointly significantly different from zero, then we say that X_t Granger-causes Y_t.

The null hypothesis (H_0) and alternative hypothesis (H_1) are:

$$H_0 : \beta_1 = \beta_2 = \cdots = \beta_q = 0 \quad \text{(No Granger causality)}$$
$$H_1 : \text{At least one } \beta_j \neq 0 \quad \text{(Granger causality exists)}.$$

The test statistic follows an F-distribution:

$$F = \frac{(SS_r - SS_{ur})/q}{SS_{ur}/(T-p-q)} \sim F(q, T-p-q), \tag{4}$$

where: SS_r is the sum of squared residuals from the restricted model (without X_t), SS_{ur} is the sum of squared residuals from the unrestricted model (with X_t), T is the number of observations. If the computed F-statistic is greater than the critical value at a given significance level (it is 0.05 in our test), we reject H_0 and conclude that X_t Granger-causes Y_t.

In this study, we apply the Granger causality test to investigate the causal relationship between heatwaves (HWs) and Rossby wave packets (RWPs) along the propagation pathway. Specifically, we examine whether the Rossby wave activity flux at each path node along the pathway has a Granger causal influence on HW or not.

Taking the starting node as an example, we first compare the time series of the surface air temperature anomaly (SATa), geopotential height anomaly at 500 hPa (z500) and the absolute amplitude of TN flux for Rossby wave activity (WAF amplitude) during heatwaves (Fig.S21). Both local maxima of the WAF amplitude and z500 precede

that of the SATa, and this holds for all four preferred pathways. Similar conclusions can be drawn from the analysis on time-lagged correlations between z500 and SATa at different path nodes (Fig.S17), where z500 precedes SATa by 1 day during the period of propagating heatwaves. This suggests that the RWP precedes HW during the period of propagating heatwaves [5, 6]. We further conduct the aforesaid Granger causality test on the paired time series of z500 and SATa, as well as WAF amplitude and SATa. The test shows that the Granger causal influence from z500 to SATa is significant, while the Granger causal influence from SATa to z500 is not significant. Similarly, it is also found that WAF amplitude unidirectionally Granger-causes SATa. Similar causal inference results are found during our analysis on other path nodes (not shown here).

Supplementary Note 4: Calculation and surrogate hypothesis test for R_{HO}

We define an index R_{HO} to assess the impact of each preferred propagation pathway on heatwave occurrences downstream [1, 2]. Taking the Asia pathway as an example, R^k_{HO} represents the probability of heatwave occurrences at the k-th path node within 7 days following a heatwave at the starting node, where $k \in [1, 6]$ represents the order of the six representative nodes along the pathway (Fig. 1).

$$R^k_{\mathrm{HO}} = \frac{N_{1|k}}{N_k} \tag{5}$$

where N_k is the total number of heatwave events at the k-th path node, and $N_{1|k}$ denotes the number of cases that a heatwave occurs at the k-th path node within 7 days following a heatwave at the starting node. Therefore, for each path node, R^k_{HO} is used to estimate the impact of the preferred propagation pathway on heatwave occurrence at that node (Fig. 5a).

To verify the significance of the preferred propagation pathway's impact on heatwave occurrences at its downstream nodes, we use surrogates to compute R_{HO} and test the null hypothesis. Specifically, when a heatwave occurs at the starting node of the Asia pathway, we estimate the probability of a heatwave occurring at different nodes within the following 7 days (i.e., R_{HO}), where each of these nodes is at the same distance from the starting node. Thus, these nodes are distributed in a circle centered on the starting node (Fig.S26): one of these nodes lies along the Asia pathway, with the probability of heatwave occurrence denoted as R_{HO}(Path); while the remaining nodes, which lie outside the Asia pathway, serve as surrogates, with their probability of heatwave occurrence denoted as R_{HO}(Non-path). Hence, the null hypothesis states that R_{HO}(Path) will not differ significantly from R_{HO}(Non-path).

We calculate R_{HO}(Non-path) values for the surrogates, and obtain the mean, 99th percentile and 1st percentile of them. The results show that R_{HO}(Path) is beyond the 99% confidence interval for the surrogates (as in Fig. 5), confirming the significant impact of preferred propagation pathway on the heatwave occurrence at its downstream nodes. Similar significance tests using surrogates were also performed for the West Europe, North America 1, and North America 2 pathways (not shown here), where the impacts of these pathways were found to be significant as well.

Supplementary Note 5: Calculating blocking index

We use Scherrer blocking index for our analysis. For each geographic grid cell λ, ϕ and day t, the meridional gradients

$$\mathrm{GHGN} = [\mathrm{Z}_{500}\,(\lambda, \phi_{\mathrm{N}}, \mathrm{t}) - \mathrm{Z}_{500}(\lambda, \phi, \mathrm{t})] \cdot g/\,(\phi_{\mathrm{N}} - \phi)$$
$$\mathrm{GHGS} = [\mathrm{Z}_{500}\,(\lambda, \phi, \mathrm{t}) - \mathrm{Z}_{500}(\lambda, \phi_{\mathrm{S}}, \mathrm{t})] \cdot g/\,(\phi - \phi_{\mathrm{S}})$$

where

$$\phi_{\mathrm{N}} = \phi + 15^{\circ}$$
$$\phi_{\mathrm{S}} = \phi - 15^{\circ}$$

Supplementary References

[1] Boers, N., Bookhagen, B., Barbosa, H.M., Marwan, N., Kurths, J., Marengo, J.A.: Prediction of extreme floods in the eastern central andes based on a complex networks approach. Nature Communications **5**, 5199 (2014)

[2] Li, K., Huang, Y., Liu, K., Wang, M., Cai, F., Zhang, J., Boers, N.: Key propagation pathways of extreme precipitation events revealed by climate networks. npj Climate and Atmospheric Science **7**(1), 165 (2024)

[3] Granger, C.W.: Investigating causal relations by econometric models and cross-spectral methods. Econometrica: journal of the Econometric Society, 424–438 (1969)

[4] Runge, J., Bathiany, S., Bollt, E., Camps-Valls, G., Coumou, D., Deyle, E., Glymour, C., Kretschmer, M., Mahecha, M.D., Muñoz-Marí, J., *et al.*: Inferring causation from time series in earth system sciences. Nature communications **10**(1), 2553 (2019)

[5] McGraw, M.C., Barnes, E.A.: Memory matters: A case for granger causality in climate variability studies. Journal of Climate **31**(8), 3289–3300 (2018)

[6] Huang, Y., Franzke, C.L., Yuan, N., Fu, Z.: Systematic identification of causal relations in high-dimensional chaotic systems: application to stratosphere-troposphere coupling. Climate Dynamics **55**, 2469–2481 (2020)
