# Supplementary material for: Evidence for preferred propagating terrestrial heatwave pathways due to Rossby wave activity
Source: Nat Commun. 2025 May 22;16:4742. doi: 10.1038/s41467-025-60104-w (PMC12095606; doi:10.1038/s41467-025-60104-w)
Supplement: Supplementary file 2 — Description of Additional Supplementary Files [file 41467_2025_60104_MOESM2_ESM.pdf]

Description of Additional Supplementary Files

File name: Supplementary Movie 1
Description: Composite animations of heatwave evolution over time for the Asian pathway under propagating (left) and standing (right) patterns. The statistical period spans from 10 days before to 10 days after the central time of the heatwave occurrence. The shading represents surface temperature anomalies, and the contours represent 500 hPa geopotential height anomalies.

File name: Supplementary Movie 2
Description: Similar to Supplementary Movie 1. Composite animations of heatwave evolution over time for the Western European pathway.

File name: Supplementary Movie 3
Description: Similar to Supplementary Movie 1. Composite animations of heatwave evolution over time for the North American pathway 1.

File name: Supplementary Movie 4
Description: Similar to Supplementary Movie 1. Composite animations of heatwave evolution over time for the North American pathway 2.
