# Supplementary figures and images for: Evidence for preferred propagating terrestrial heatwave pathways due to Rossby wave activity
Source: Nat Commun. 2025 May 22;16:4742. doi: 10.1038/s41467-025-60104-w (PMC12095606; doi:10.1038/s41467-025-60104-w)

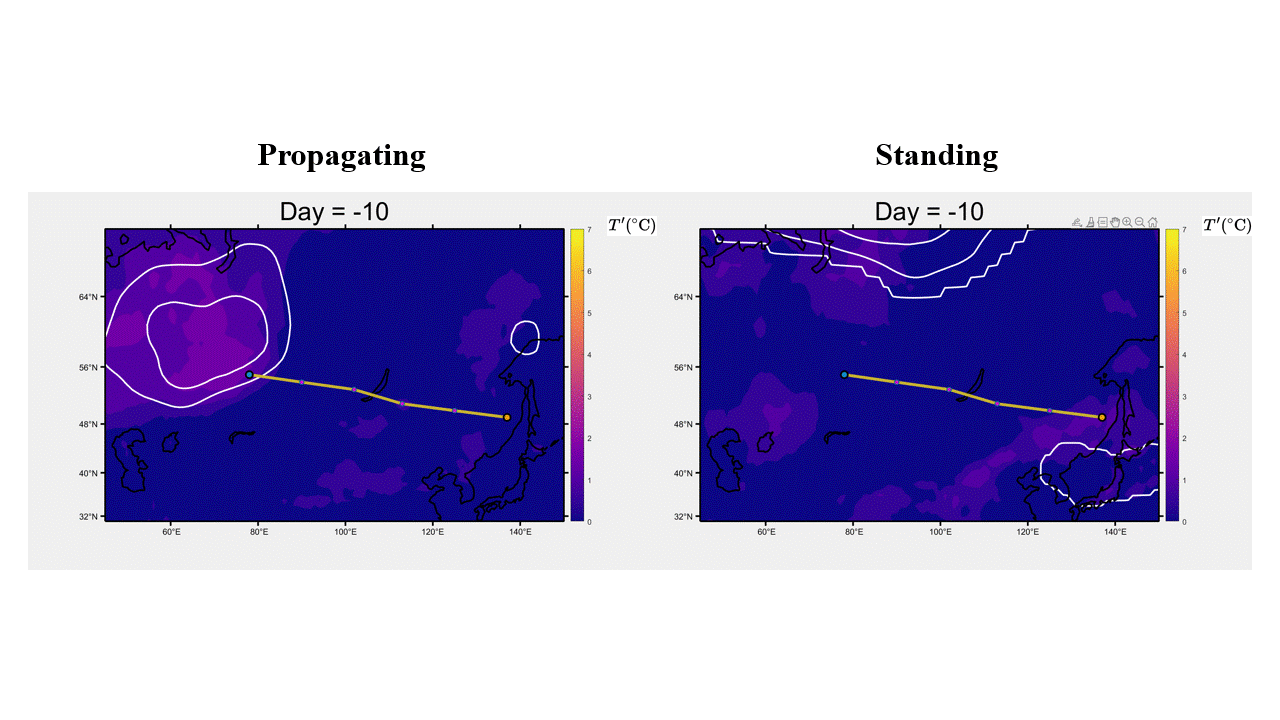

Supplement: Supplementary file 3 — Supplementary Movie 1 [file 41467_2025_60104_MOESM3_ESM.gif]

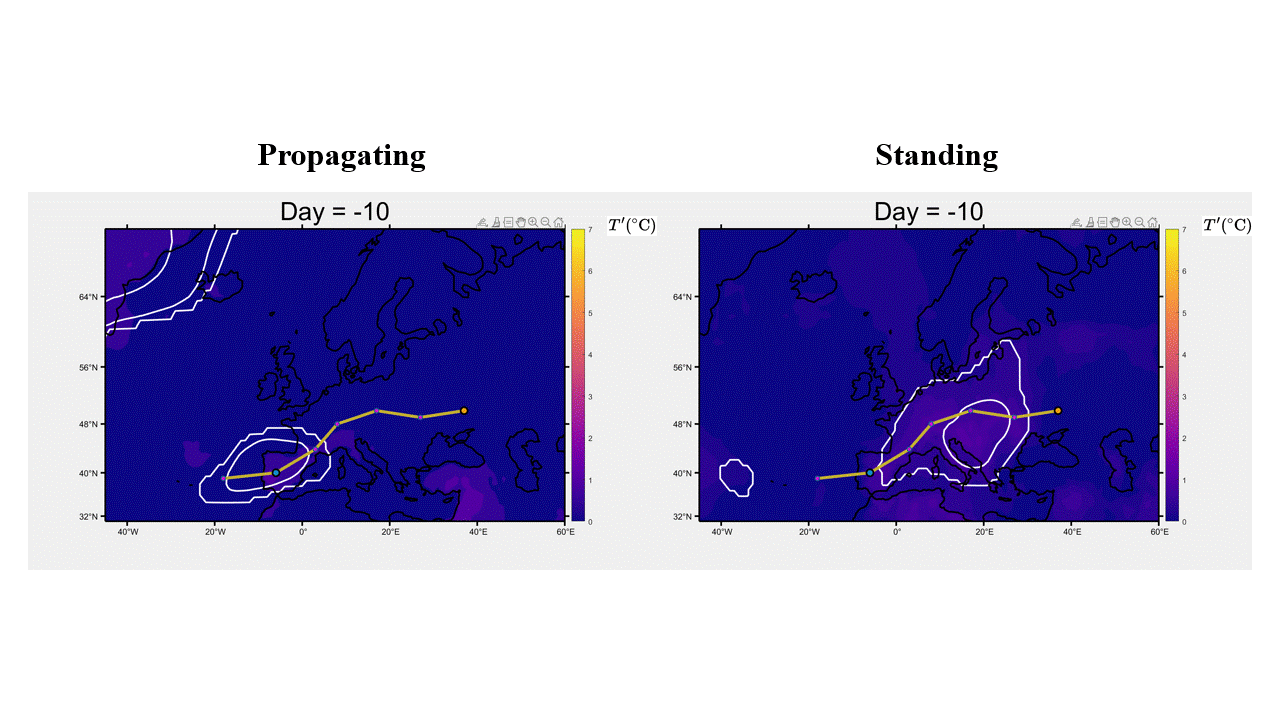

Supplement: Supplementary file 4 — Supplementary Movie 2 [file 41467_2025_60104_MOESM4_ESM.gif]

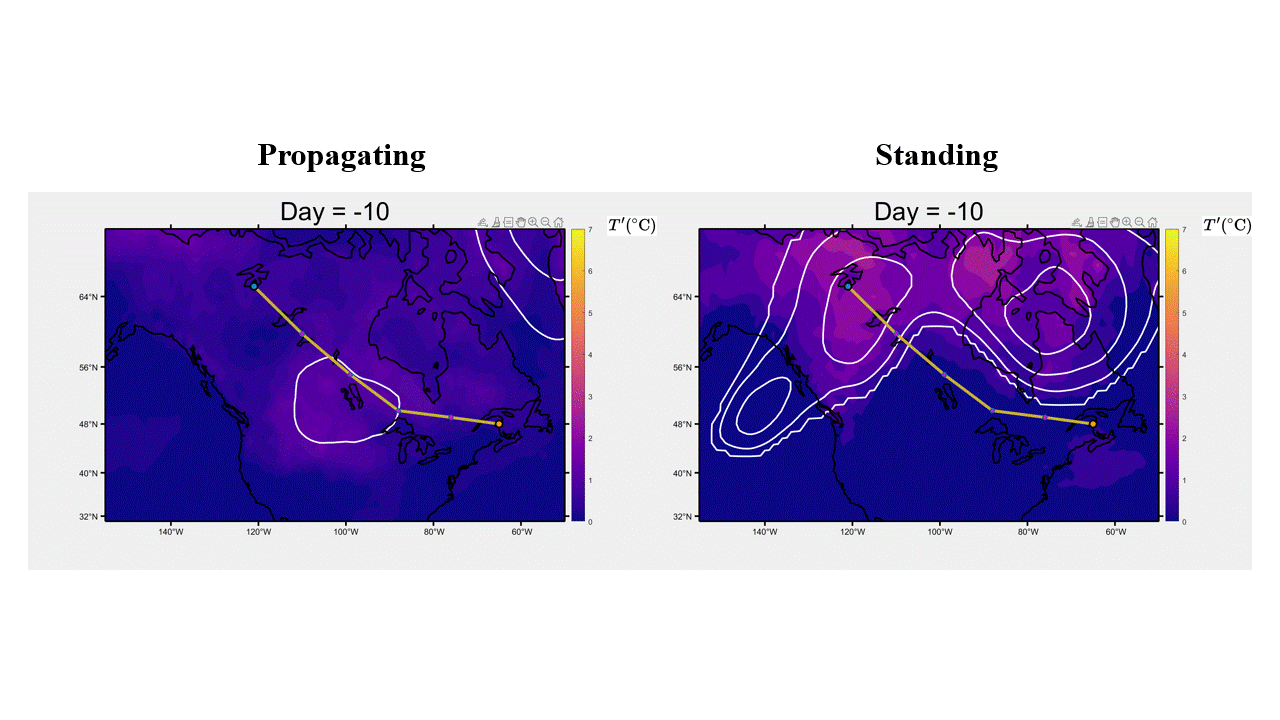

Supplement: Supplementary file 5 — Supplementary Movie 3 [file 41467_2025_60104_MOESM5_ESM.gif]

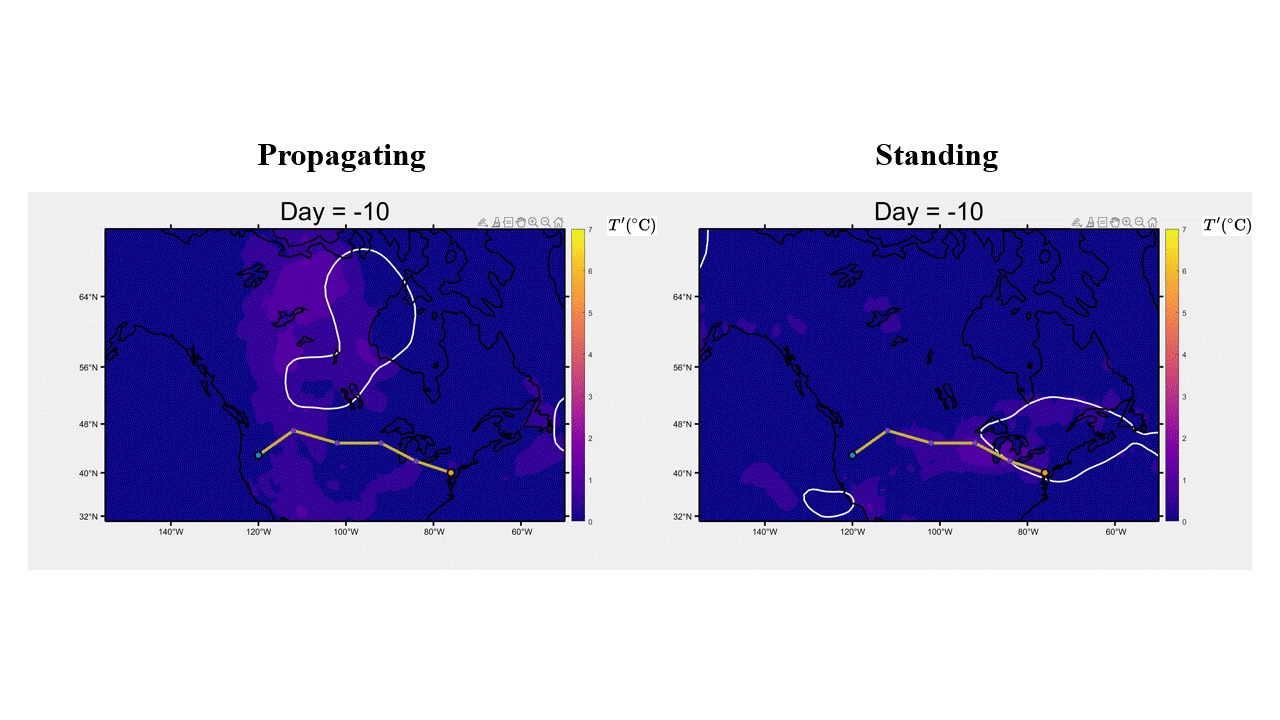

Supplement: Supplementary file 6 — Supplementary Movie 4 [file 41467_2025_60104_MOESM6_ESM.gif]
